# Explore the Underlying Mechanism Between Atopic Dermatitis and Major Depressive Disorder

**DOI:** 10.3389/fgene.2021.640951

**Published:** 2021-05-28

**Authors:** Tao Yang, Xuehua Huang, Jiajun Xu, Mingjing Situ, Qingqing Xiao, Kamil Can Kural, Yan Kang

**Affiliations:** ^1^Mental Health Center, West China Hospital, Sichuan University, Chengdu, China; ^2^School of Systems Biology, George Mason University, Fairfax, VA, United States; ^3^Human Biochemical Genetics Section, National Institutes of Health, Bethesda, MD, United States

**Keywords:** atopic dermatitis, major depressive disorder, literature-based data mining, mega-analysis, multiple linear regression analysis, gene set enrichment analysis, protein-protein interaction analysis

## Abstract

Adult patients with atopic dermatitis (AD) present relatively higher rates of major depressive disorder (MDD). However, the underlying mechanism is largely unknown. Here, we first conducted a systematic literature-based data mining to identify entities linking AD and MDD, including proteins, cells, functional classes, and small molecules. Then we conducted an AD-RNA expression data-based mega-analysis to test the expression variance of the genes that were regulators of MDD. After that, a Fisher Exact test-based pathway enrichment analysis (PEA) was performed to explore the AD-driven MDD-genetic regulators’ functionality. We identified 22 AD-driven entities that were up-stream MDD regulators, including 11 genes, seven small molecules, three functional classes, and one cell. AD could exert a promoting effect on the development of MDD. Four of the 11 genes demonstrated significant expression changes in AD patients in favor of the development of MDD. PEA results showed that AD mainly drives cytokine/chemokine regulation and neuroinflammatory response-related pathways to influence the pathological development of MDD. Our results supported the promotion role of AD in the pathological development of MDD, including the regulation of multiple genetic regulators of MDD involved in cytokine/chemokine regulation and inflammatory response.

## Introduction

Atopic dermatitis (AD), also known as atopic eczema, is long-term inflammation of the skin. AD is characterized by pruritic erythema lesions, commonly located in curved areas, face, and hands (Tollefson et al., 2014). AD is not fatal, but the accompanying irritation, itching, and other symptoms can cause sleep disturbance and heat sensation, thereby increasing AD patients’ psychological burden (Absolon et al., 1997). For example, a case and control study conducted in school-aged children indicated that AD cases are at high risk of developing psychological difficulties (Absolon et al., 1997).

The relationship between AD symptoms and the prevalence of depression was reported, such as major depressive disorder (Hashiro and Okumura, 1997; Gupta and Gupta, 1998; Sanna et al., 2014; Cheng et al., 2015; Lee and Shin, 2017). Major depressive disorder (MDD), also known as depression, is a mental disorder characterized by at least 2 weeks of pervasive low mood. It has been reported that adult patients with AD had relatively higher rates of clinical depression, antidepressant use, and suicidality (Cheng and Silverberg, 2019). Approximately 1 in 3 adults with AD met diagnostic criteria for MDD (Blaiss, 2019). Besides, the associations’ strengths varied depending on the severity of AD symptoms (Hashiro and Okumura, 1994; Sanna et al., 2014). Hashiro et al. reported that patients with AD increased the scores according to the severity of their AD on a psychological disturbances scale (Hashiro and Okumura, 1994). Other cross-sectional, population-based studies found consistent results (Sanna et al., 2014).

Although there have been concerns about MDD among patients with AD, the underlying mechanism that AD promotes the development of MDD is largely unknown. Here, we hypothesize that the AD-driven molecule changes in patients may promote the development of MDD. We first conducted a systematic literature data mining to uncover connections between AD and MDD at different levels. Then, we performed a mega-analysis to test the expression levels of MDD regulators (genes) in the case of AD. At last, a gene set enrichment analysis was conducted to explore the functional profile of the genetic regulators of MDD that was also driven by AD. Our results enabled us to uncover functional networks and pathways that may partially explain AD’s influences on MDD.

## Materials and Methods

**Figure 1:**
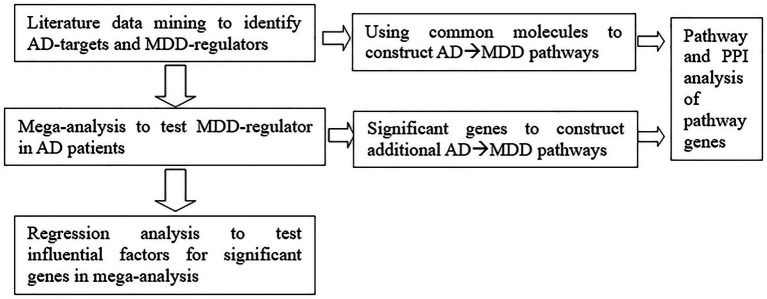
Workflow diagram.

To explore the underlying mechanism of increased MDD risk for patients with AD, we first conducted a systematic literature-based data mining to identify entities linking AD and MDD, including proteins, cells, functional classes, and small molecules. Then we conducted an AD-RNA expression data-based mega-analysis to test the expression variance of the genes that were regulators of MDD. After that, we performed pathway analysis to explore the functionality of the AD-driven MDD-genetic regulators. We provide the workflow diagram of this study in Figure 1.

### Literature-Based Data Mining to Identify AD-MDD Linkage

Predominant molecular pathways and networks are becoming a common aid in inferring novel pathophysiological insights from changes in the levels of various biomolecules profiled in a high-throughput fashion (Ochs, 2010). Here, assisted by the tool Pathway Studio^1^ (version 12.3.0.16), we conducted systematic literature-data mining to uncover biological factors linking AD to MDD. Pathway Studio contains a literature-based database covering over 24 million PubMed abstracts and over 3.5 million Elsevier and 3rd part full-text papers. It contains 13 types of entities, including 138,106 genes, 1,053,259 small molecules, 5,489 functional classes, and 4,155 cells. There were about 12.8 million relations identified among these entities, with each of them being supported by one or more references. Here, we identified the biological factors influencing AD and also were regulators of MDD, and provided the relevant information for the references supporting each of these relations in Supplementary Material AD_MDD→Ref4AD_MDD. Specifically, we first identified the cells, functional classes, small molecules, and genes that regulate MDD by employing the network building function of Pathway Studio. These molecules were identified through literature data mining to show the relationship with MDD. Details regarding the network building function of Pathway Studio were described in https://supportcontent.elsevier.com/Support%20Hub/Pathway%20Studio/7680_Network_Builder.pdf. Then, following the same process, we identified the items that were regulated by AD. All relationships without polarity (positive or negative) were filtered out. Then the overlapped items were used to construct the network that connects AD and MDD. The definition of different entities and relationships within the network were presented in https://supportcontent.elsevier.com/RightNow%20Next%20Gen/Pathway%20Studio/2683_PS_QuickStartGuide_2020.pdf.

### Gene Expression Data Selected for Mega-Analysis

Following the initial search with ‘atopic dermatitis’, 103 microarray expression datasets were identified on gene expression omnibus (GEO).^2^Subsequently, the following criteria were applied:

1) The organism used in the study was *Homo sapiens*.

2) The data type was microarray expression profiling.

3) The studies were limited to a comparison between AD and healthy controls.

4) The original data and the corresponding format file were downloadable.

A total of nine datasets satisfied the mega-analysis’ inclusion criteria. We provided the information of these datasets in Table 1.

**Table 1:** The nine atopic dermatitis expression datasets employed for mega-analysis.

GEO ID	#Control/#case	Country	Study age (year)	Sample organism	Patient sample source	Disease name	Platform
GSE5667	5/12	United States	15	*Homo sapiens*	lesional and nonlesional skin	atopic dermatitis	GPL96
GSE6012	10/10	Sweden	15	Homo sapiens	lesional skin	atopic eczema	GPL96
GSE16161	9/9	United States	12	Homo sapiens	lesional skin	atopic dermatitis	GPL570
GSE26952	7/5	United States	10	Homo sapiens	nonlesional skin	atopic dermatitis, psoriasis	GPL2700
GSE32924	8/25	United States	10	Homo sapiens	lesional and nonlesional skin	atopic dermatitis	GPL570
GSE60709	21/12	Germany	7	Homo sapiens	lesional and nonlesional skin	atopic dermatitis	GPL6947
GSE116486	18/28	United States	3	Homo sapiens	blood	atopic dermatitis	GPL570
GSE120721	22/30	United States	3	Homo sapiens	lesional and nonlesional skin	atopic dermatitis	GPL570
GSE153007	5/24	United States	1	Homo sapiens	lesional skin	atopic dermatitis	GPL6480

### Mega-Analysis Models

For the 203 genes that were upstream regulators of MDD (see Supplementary Material AD_MDD→MDD Regulators), we performed a partial mega-analysis (Dong et al., 2020) using the nine RNA expression data presented in Table 1. The log2 fold-change (LFC) of the gene expression level was used to indicate the effect size. Both fixed-effect and random-effects models were employed to investigate and compare the effect size (Borenstein et al., 2010). Mega-analysis is a one-stage approach that analyzes all individual datasets in one statistical model to estimate an overall effect, and meta-analysis is a two-stage approach, analyzing the individual datasets from each study separately to obtain summary data and then using standard meta-analytical techniques, such as a random-effects meta-analysis model (Boedhoe et al., 2019). In this study, the term “mega-analysis” instead of “meta-analysis” was used to reflect the fact that the effect size of each gene was calculated from the original data using one statistical analysis workflow rather than extracted summary results from previous studies.

The heterogeneity of the mega-analysis was analyzed to study the variance within and between different studies. In the case that the total variance (Q) was equal to or smaller than the expected between-study variance (df), the within-study variance percentage (ISq) = 100% * (Q-df)/Q was set at 0, and a fixed-effect model was selected for the mega-analysis. Otherwise, a random-effects model was selected. Q-p represents the probability that the total variance was only due to within-study variance. The current study presented all the mega-analysis results identified in AD_MDD→Mega-analysis. All analyses were performed using Matlab (version R2017a^3^). Mega-analysis package was developed according to the algorithm developed in (Borenstein et al., 2010).

Among the 203 genes tested in mega-analysis, those showing significant expression changes in AD patients were identified, and their relationship with MDD was studied through Pathway Studio-assisted literature data mining. The results will be used to construct an additional functional network that may add more insights into the ADMDD regulation mechanism.

### Multiple Linear Regression Analysis

A multiple linear regression (MLR) model was employed to investigate the possible influence of sample size, country of origin, and study date on the gene expression in the case of AD. *p*-values were reported for each of these factors.

### Pathway Enrichment Analysis and Protein-Protein Interaction Analysis

To test the functional profile of the genes involved in the AD-driven MDD genetic regulators, a Pathway Enrichment Analysis (PEA) was conducted using Pathway Studio (version 12.1.0.16)^4^ against Gene Ontology (GO).^5^ Fisher Exact test *p*-value was used to determine the overlap between an input gene list and a GO term.^6^ The PEA results were reported with enrichment p-value corrected using the original Benjamini and Hochberg false discovery ratio (FDR) procedure (Benjamini and Hochberg, 1995). Based on the PEA results, a protein-protein interaction (PPI) network was constructed. Two genes were recognized as connected if they were identified to play roles within at least one common pathway or functional group. We also employed Human Reference Interactome (HuRI) to explore the physical interactions of proteins we found relevant with mega-analysis.

## Results

### The Common Genes Between AD and MDD

**Figure 2:**
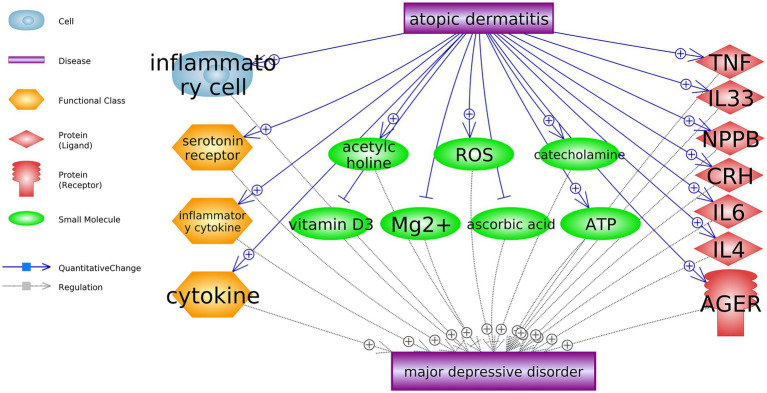
Atopic dermatitis driven biology entities that promote the development of major depressive disorder.

Through large-scale literature data mining, we identified 18 AD-driven entities that were up-stream MDD regulators, as shown in Figure 2. These 18 entities include seven genes, seven small molecules, three functional classes, and one cell. Three of these entities were MDD inhibitors, including vitamin D3, Mg2+, and ascorbic acid. AD could promote the development of MDD by deactivating these AD inhibitors. On the other hand, AD could activate 15 MDD promoters, which may exert stronger promoting effects on the development of MDD. In the Supplementary Material AD_MDD→Ref4AD_MDD, the reference-related information was provided for each of these relationships in Figure 2, including titles and sentences where these relationships were identified.

### Mega-Analysis Results

Mega-analysis showed six genes presented significant expression changes in AD patients, as shown in Table 2. Moreover, the MLR results in Table 2 suggested that the sample size was a significant factor to influence the expression levels of one gene (ISG15). However, the three parameters (country, sample size, and study age) presented no significant influence on most of these six genes.

**Table 2:** Significant genes in atopic dermatitis RNA expression-based mega-analysis.

	Mega-analysis results				MLR analysis results (*p*-value)		
Gene name	Random effects model (Yes = 1, No = 0)	N Study	Effect Size	*p*-value	N Sample	Country	Study age
CCL22	0	4	2.765	2.10 E-3	0.17	0.26	0.066
ISG15	0	4	1.452	2.54E-3	0.06	0.61	0.014
CC2D1A	0	4	1.009	7.42E-05	0.81	0.58	0.21
USP46	0	4	−1.07	1.72E-08	0.97	0.18	0.98
BMP7	0	4	−1.17	0	0.56	0.14	0.13
AR	1	4	−1.2	8.16E-4	0.30	0.32	0.44

**Figure 3:**
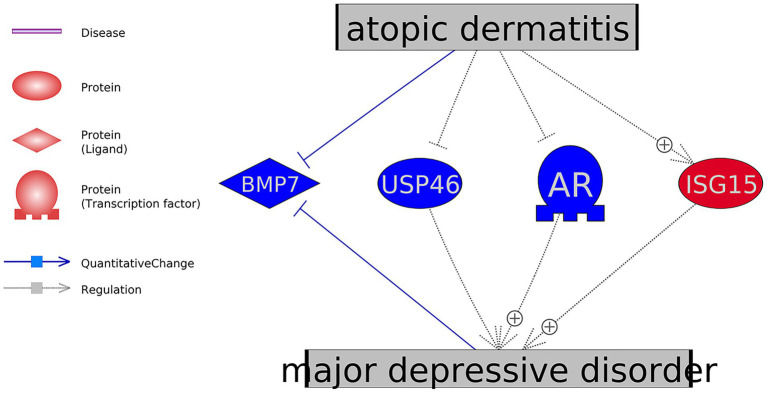
Mega-analysis uncovered four genes showed significant expression changes in atopic dermatitis in favor of the development of major depressive disorder.

Pathway Studio assisted literature data mining results showed that, among these genes that presented the significant expression variations in AD, the expression changes of four genes favored the development of MDD, as shown in Figure 3. The references supporting the relation between MDD and the four genes in Figure 3 were presented in AD_MDD→Ref4AD_MDD1. The relationships between AD and these four genes were built based on mega-analysis results.

### PEA Results and Network Analysis

**Figure 4:**
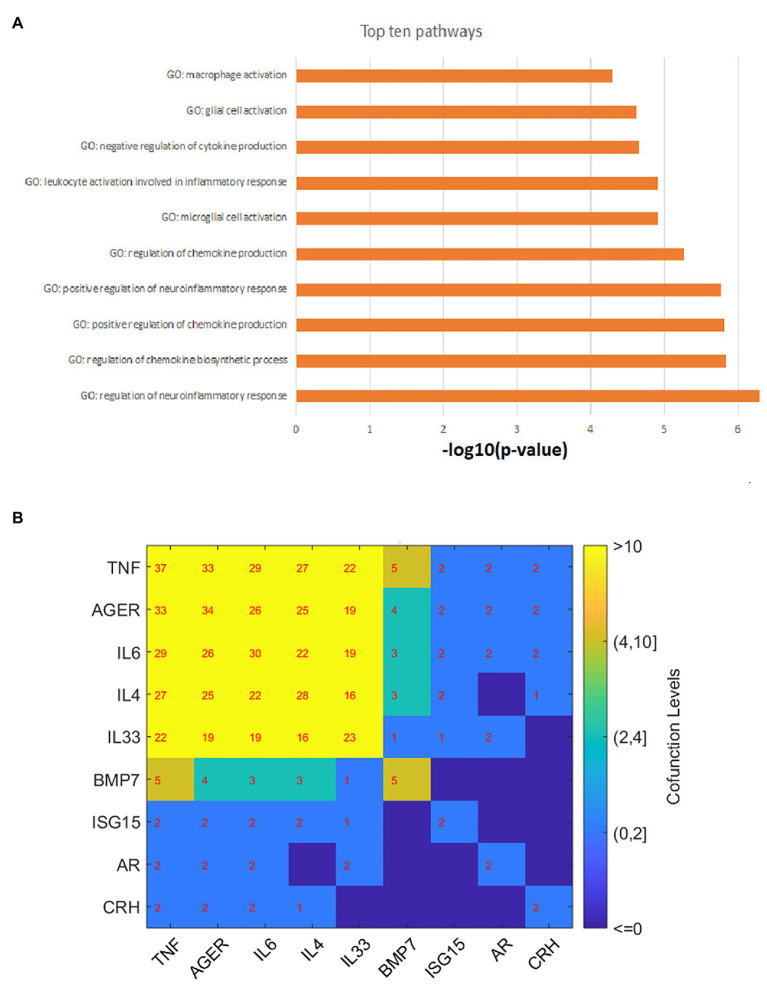
Gene set enrichment analysis results and gene–gene interaction network. **(A)** The top 10 GO terms enriched by 11 atopic dermatitis-driven genes promoting the development of major depressive disorder. **(B)** The pathway-based gene-gene interaction network. The number within each cell represents the number of pathways two genes shared.

The PEA was conducted to test the functional profile of the 11 genes identified in the AD-driven pathways in Figures 2, 3. The 10 most significantly enriched pathways (*p*-value < 5.18E-5, *q* = 0.001 for FDR) are presented in Figure 4A. The full 38 pathways/gene sets enriched with nine genes (*p*-value < 0.001) have been listed in Supplementary Material AD_MDD→PEA.

As shown in Figure 4A, most of these pathways/gene sets were related to cytokine/chemokine regulation and inflammatory response. Moreover, five genes were enriched in three immune system-related pathways [*p*-value: (0.00011, 0.00096); GO ID: 0002822; 0002819; and 0002700]. These pathways were previously implicated in the development of MDD (Kim et al., 2012; Miller and Raison, 2016; Money et al., 2016). For detailed information on these significantly enriched pathways, please refer to Supplementary Material AD_MDD→PEA.

Based on the PEA results, we build a gene-gene interaction (GGI) network, as shown in Figure 4B. Results showed that most of these genes play roles in multiple common pathways, indicating the functional linkage among them in the regulations of MDD.

**Figure 5:**
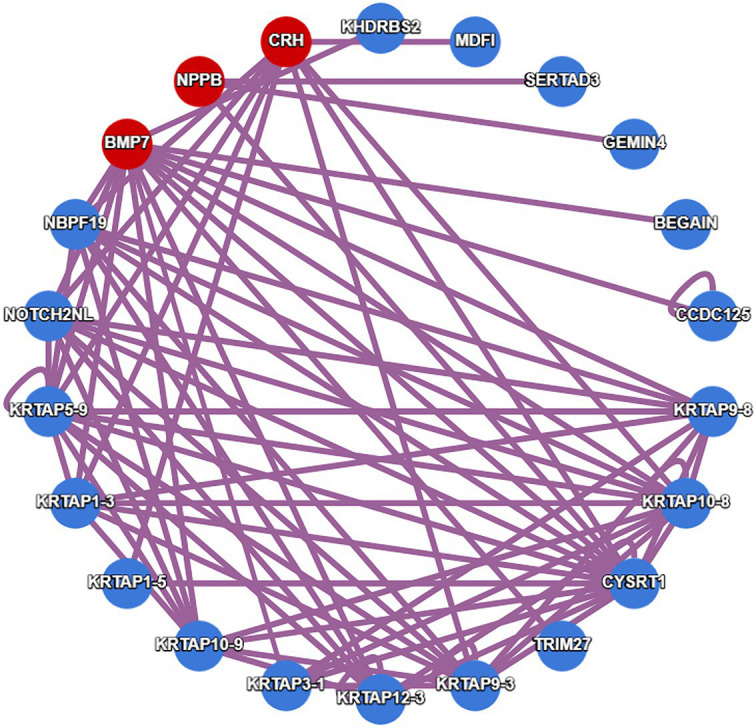
Protein Interaction network from human reference interactome (HuRI).

We also used HuRI (Human Reference Interactome) to explore the physical interactions of genes identified in both function networks (Figures 2, 3), as shown in Figure 5. Results showed that genes CRH, NPPB, and BMP7 were physically linked to common targets, which supported the potential functional connection among the three genes.

## Discussion

In this study, we integrated literature data mining results and RNA expression data-based mega-analysis results to construct functional networks revealing potential mechanisms underlying the MDD promoting-effect of AD. Our results showed that AD could influence multiple MDD regulators at different levels and drive cytokine/chemokine regulation and inflammatory response-related pathways that play important roles in the development of MDD.

Literature data mining showed that AD could impact seven genes, seven small molecules, three functional classes, and one cell, which are upstream regulators of MDD. AD patients present accumulated inflammatory cells (Sung et al., 2011), which may induce depression (Qian et al., 2019). At the functional class level, AD has been shown to present over-expressed cytokines, serotonin receptors, and inflammatory cytokines (Schmid-Ott et al., 2001; Kitaichi et al., 2006; Kim et al., 2013), which play critical roles in the pathogenesis of MDD (González-Maeso et al., 2002; Miller, 2009). The top 10 GO terms enriched by 11 atopic dermatitis-driven genes promote major depressive disorder development. By the pathway-based gene-gene interaction network, the number within each cell represents the number of pathways two genes shared.

AD patients demonstrated increased acetylcholine levels (Profita et al., 2008), while clinical studies showed that increased central acetylcholine could lead to a depressed mood (Dulawa and Janowsky, 2019). AD patients also present increased levels of reactive oxygen species (ROS; Choi et al., 2010), catecholamine (Buske-Kirschbaum et al., 2002), and arabinosyladenine triphosphate (ATP; Blunder et al., 2018), which have pivotal roles in the biological mechanisms of MDD (Xu et al., 2011; Cao et al., 2013; Dhingra and Bansal, 2015). On the other hand, AD patients have lower levels of vitamin C, vitamin D3, and Mg2+, which were shown to have therapeutic value in MDD (Bodnar and Wisner, 2005; Ghasemi et al., 2014; Smolders et al., 2017). These findings suggested that AD might promote the development of MDD by regulating multiple small molecules in the human body.

At the genetic level, there were 11 genes which have been shown as AD-driven regulators of MDD, including eight MDD promoters (TNF, IL33, NPPB, CRH, IL6, IL4, AGER, and ISG15) and three MDD inhibitors (BMP7, USP46, and AR), as shown in Figures 2, 3. Specifically, four of these 11 genes presented significant expression changes in AD cases (mega-analysis using nine AD RNA expression datasets; see Table 2), including ISG15, BMP7, USP46, and AR. These results support the relationship between these four genes, AD, and MDD, suggesting that AD might regulate the expression levels of these four genes to influence the pathological development of MDD. However, considering the expression data sources were from skin and blood, a direct experiment should be conducted to test the expression level connection between AD and MDD (e.g., only using data collected from peripheral blood).

We also noted that the expression changes of the other two genes (CCL22 and CC2D1A) in AD patients might not favor the development of MDD. Therefore, further study is needed to test the genetic regulators of MDD in AD patients.

PEA results showed that most of the AD-driven genes regulating MDD were enriched within cytokine/chemokine regulation and neuroinflammatory response-related pathways, as shown in Figure 4 and Supplementary Material AD_MDD→PEA. AD has been shown to present over-expressed cytokines/chemokine (Schmid-Ott et al., 2001), which play critical roles in the pathogenesis of MDD (Miller, 2009). Several main findings supported the neuroinflammatory theory of MDD, which hypothesizes that cerebral inflammation is crucial to the development of depression and other neuropsychiatric diseases (Duque and Fricchione, 2019). Elevated microglia activation and neuroinflammatory response have been observed in the brains of AD by using a mice model (Park et al., 2020), which may add to the explanation of the AD-promoting effect on the development of MDD. Moreover, three out of the 11 AD-driven MDD regulating genes (Figures 2, 3) presented physical linkage to common targets, supporting their functional connection.

## Conclusion

Our study indicated that AD might drive multiple molecules that were implicated as upstream regulators of MDD, including genes involved in cytokine/chemokine regulation and inflammatory response.

## Data Availability Statement

The original contributions presented in the study are included in the article/Supplementary Material; further inquiries can be directed to the corresponding author.

## Author Contributions

TY and XH developed the study design, analyzed the data, and wrote the original paper. All authors contributed to the article and approved the submitted version.

### Conflict of Interest

The authors declare that the research was conducted in the absence of any commercial or financial relationships that could be construed as a potential conflict of interest.

## Footnotes

^1^www.pathwaystudio.com

^2^https://www.ncbi.nlm.nih.gov/geo/

^3^https://www.mathworks.com/products/matlab.html

^4^www.pathwaystudio.com

^5^http://geneontology.org

^6^https://www.pathwaycommons.org/guide/primers/statistics/fishers_exact_test/
